# A remarkably diverse and well-organized virus community in a filter-feeding oyster

**DOI:** 10.1186/s40168-022-01431-8

**Published:** 2023-01-07

**Authors:** Jing-Zhe Jiang, Yi-Fei Fang, Hong-Ying Wei, Peng Zhu, Min Liu, Wen-Guang Yuan, Li-Ling Yang, Ying-Xiang Guo, Tao Jin, Mang Shi, Tuo Yao, Jie Lu, Ling-Tong Ye, Shao-Kun Shi, Meng Wang, Ming Duan, Dian-Chang Zhang

**Affiliations:** 1 Key Laboratory of South China Sea Fishery Resources Exploitation and Utilization, Ministry of Agriculture and Rural Affairs, South China Sea Fisheries Research Institute, Chinese Academy of Fishery Sciences, Guangzhou, 510300 Guangdong China; 2 College of Fisheries and Life Science, Shanghai Ocean University, Shanghai, 201306 China; 3 Guangdong Province Key Laboratory for Biotechnology Drug Candidates, School of Biosciences and Biopharmaceutics, Guangdong Pharmaceutical University, Guangzhou, 510006 Guangdong China; 4 Tianjin Agricultural University, Tianjin, 300384 China; 5 Present Address: State Key Laboratory of Freshwater Ecology and Biotechnology, Institute of Hydrobiology, Chinese Academy of Sciences, Wuhan, 430072 China Hubei; 6 Present Address: Guangdong Magigene Biotechnology Co Ltd, Guangzhou, 510000 Guangdong China; 7 School of Medicine, Sun Yat-Sen University, Shenzhen, 518107 Guangdong China; 8 Shenzhen Fisheries Development Research Center, Shenzhen, 518067 Guangdong China; 9 Bureau of Agriculture and Rural Affairs of Conghua District, Guangzhou, 510925 Guangdong China; 10 Shanghai Majorbio Bio-Pharm Technology Co Ltd, Shanghai, 201203 China

**Keywords:** *Crassostrea hongkongensis*, Bivalve, Mollusk, Metagenome, Circovirus, Viral-like particle enrichment, Mussel Watch, Multiple displacement amplification

## Abstract

**Background:**

Viruses play critical roles in the marine environment because of their interactions with an extremely broad range of potential hosts. Many studies of viruses in seawater have been published, but viruses that inhabit marine animals have been largely neglected. Oysters are keystone species in coastal ecosystems, yet as filter-feeding bivalves with very large roosting numbers and species co-habitation, it is not clear what role they play in marine virus transmission and coastal microbiome regulation.

**Results:**

Here, we report a Dataset of Oyster Virome (DOV) that contains 728,784 nonredundant viral operational taxonomic unit contigs (≥ 800 bp) and 3473 high-quality viral genomes, enabling the first comprehensive overview of both DNA and RNA viral communities in the oyster *Crassostrea hongkongensis*. We discovered tremendous diversity among novel viruses that inhabit this oyster using multiple approaches, including reads recruitment, viral operational taxonomic units, and high-quality virus genomes. Our results show that these viruses are very different from viruses in the oceans or other habitats. In particular, the high diversity of novel circoviruses that we found in the oysters indicates that oysters may be potential hotspots for circoviruses. Notably, the viruses that were enriched in oysters are not random but are well-organized communities that can respond to changes in the health state of the host and the external environment at both compositional and functional levels.

**Conclusions:**

In this study, we generated a first “knowledge landscape” of the oyster virome, which has increased the number of known oyster-related viruses by tens of thousands. Our results suggest that oysters provide a unique habitat that is different from that of seawater, and highlight the importance of filter-feeding bivalves for marine virus exploration as well as their essential but still invisible roles in regulating marine ecosystems.

Video Abstract

**Supplementary Information:**

The online version contains supplementary material available at 10.1186/s40168-022-01431-8.

## Background

As the most abundant biological entities on Earth, viruses can infect organisms from every phylum. They play critical roles in host mortality, metabolism, physiology, and evolution, impacting marine biogeochemical cycling and shaping the Earth’s microbiomes [27, 104, 92]. Culture-independent next-generation sequencing technologies have recently been used to explore the tremendous diversity of the virosphere from multiple samples [13, 59, 60, 66, 84, 85]. Among the findings, progress in the discovery of marine viruses (mainly phages of marine bacteria) is particularly impressive [12], including the creation of a global ocean DNA virome 2.0 (GOV 2.0) dataset, which contains 195,728 viral populations detected from 145 seawater samples collected worldwide [31].

Many studies have focused on free viruses in seawater, whereas viruses in marine animals have been largely neglected. Marine animals are teeming with viruses that inhabit hosts’ surfaces, body spaces, and blood [82]. Virome of marine animals form connections with their host, which is vital to the interaction of the microbe community both in and outside the host’s body [2, 30].

Bivalves of the phylum Mollusca (i.e., oysters, mussels, scallops, and clams) represent the largest number of described marine animal species and they are known to play vital roles in the functioning of marine ecosystems. Many bivalves are important fishery and aquaculture species as well as models for studying ocean acidification, biomineralization, and adaptation to coastal environments under climate change [52, 73, 106]. Some sedentary bivalves, such as oysters and mussels, impose a stabilizing and enduring ecological effect on a given area. However, their population characteristics, which include high roost numbers and species co-habitation, provide ideal conditions for the transmission of viruses with the water flow. Importantly, as filter-feeding animals, bivalves can draw up to 5 L of water per hour through their gills and thereby concentrate suspended microbes and particles by factors of a thousand to a hundred-thousand times the concentrations found in seawater [5, 65]. Indeed, the enrichment of human enteric viruses [61] and mimiviruses [1] in oyster gill or gut tissues is clearly an effect of their filter-feeding habit.

Bivalves have a semi-open circulatory system and lack body segmentation,their hemolymph is pumped into a cavity (hemocoel) and the material in it is directly exchanged between the blood and body cells. Consequently, it is interesting to speculate on the microbial communities present in bivalves. Previous studies have shown that the microbiota in oysters is mainly affected by the external environment and by disturbances [55, 56, 63, 99], although the internal microbial community can differ significantly from the microbiota in the ambient water. This indicates that the internal environment of the oyster has a selective effect on the microbiota that it hosts [88,54]. To date, few studies have reported on the viral microbial community in oysters [22]. Whether bivalves provide a similar environment or a unique habitat for marine viruses and whether bivalves spread viruses and regulate coastal microbial communities are important questions yet to be answered.

Oysters of the family Ostreidae are widely distributed in the intertidal zone globally and are possibly the most highly produced seafood in the world. China is the largest producer of oysters, accounting for 85.3% of the world’s total production (FAO, 2019). Here, we report an extensive Dataset of Oyster Virome (DOV) that consists of 54 sequencing libraries from different tissues, sampling sites, and sampling times of *Crassostrea hongkongensis*, the most farmed species of oyster along the south coast of China. We used virus-like particle (VLP) enrichment and targeted amplification strategies and thereby built a ‘knowledge landscape’ of the oyster virome community, its function, and the factors influencing both RNA and DNA viruses, which provides a good foundation to address questions about the connections between bivalves and marine viruses.

## Material and methods

### Oyster sampling

**Fig. 1:**
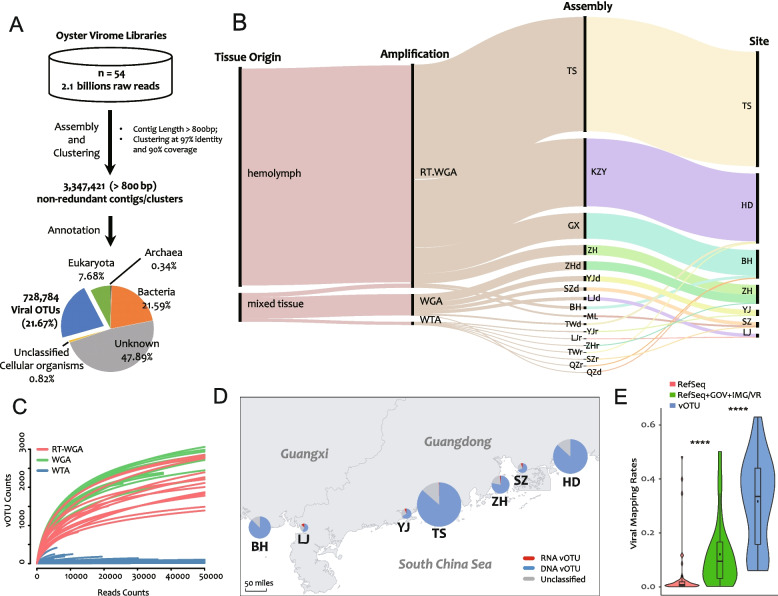
Overview of the Dataset of Oyster Virome (DOV). **A** De novo assembly and annotation pipeline. **B** Sankey diagram of the relationship among different batches and groups. The heights of the black vertical bars proportionally represent the number of viral contigs (vOTUs) assembled under the each group. **C** Rarefaction curves of the oyster viromic libraries. RT-WGA, reverse transcription and whole genome amplification; WGA, whole genome amplification; WTA whole transcriptome amplification. **D** Sampling site distribution map and the number of detected vOTUs from each site. The radius of the pie chart indicates the number of DNA, RNA, and unclassified vOTUs. **E** Mapping rates of viral reads in total clean reads. RefSeq, NCBI viral RefSeq genomes (release March 2021); GOV (release July 2020), Global Ocean Virome dataset; IMG/VR (release January 2018), a database of cultured and uncultured DNA viruses and retroviruses maintained by the Joint Genome Institute; vOTU, de novo assembled vOTUs in the DOV. “****” indicates *p* < 0.0001 (Student’s *t*-test between the three mapping rates)

The oyster samples in this study were all adults of *C. hongkongensis* and the sample collection spanned 5 years, from June 2014 to July 2019. We divided the samples into nine *time* batches according to the chronological order of collection (Table S1: Time_Batch_ID, Sampling_Date). In addition, the samples were divided into four other groups. *Amplification* groups were based on the amplification method: whole genome amplification (WGA), whole transcriptome amplification (WTA), reverse transcription and WGA (RT-WGA), or double-stranded DNA (dsDNA) (Table S1: Amplification_Method). *Tissue* groups were based on the tissue origin (i.e., mixed tissues and hemolymph of adults) (Table S1: Tissue_Origin). *Site* groups were based on the sampling site (BH, HD, LJ, SZ, TS, YJ, and ZH) (Fig. 1D) (Table S1: Sampling_Site). Finally, *status* groups were based on the health status of the oyster (i.e., apparently healthy or moribund) (Table S1: Health_Status). The designation “healthy” denotes that there was no large-scale death of farmed oysters before or after sampling and that normal and fleshy individuals were collected. The designation “moribund” indicates that large-scale mortality was taking place at the time of sampling, and consequently, surviving but moribund individuals were collected. In total, we constructed 54 sequencing libraries (Table S1: Library_ID) with 35 samples (Table S1: Sample_ID).

Time batch 1 (dCh) comprised dying animals collected from an oyster farming area at Beihai (BH), Guangxi Province, in June 2014. Time batch 2 included 3 samples (ChYJa–c) collected from an oyster farming area at Yangjiang (YJ), Guangdong Province, in September 2015. Time batch 3 comprised 12 samples (QZa–c, TWa–c, ZHa–c, and LJa–c) that were separately collected from oyster farming areas in the Qinzhou area (QZ) of BH, the Tanwei area (TW) of Huidong (HD), and at Zhuhai (ZH) and Lianjiang (LJ) in Guangdong Province in November 2015. Time batch 4 comprised 3 samples (SZa–c) collected from the Shenzhen (SZ) oyster farming area in Guangdong Province in April 2016. Time batch 5 comprised 3 samples (ML-1–3) collected at SZ in May 2016. Time batch 6 contained 2 moribund samples (BHos1–2) collected in BH in July 2016. Time batch 7 comprised 9 samples (GX, K1ZY, K2ZY, T2S, T4S, T5S, T6S, T8S, and ZH) which were separately collected from BH, Kaozhouyang (K#ZY) of Huidong (HD), Taishan (T#S), and ZH in Guangdong Province in May 2017; of these, samples K1ZY, K2ZY, and T8S were healthy, and the others were moribund. Time batch 8 (os) were oysters collected in July 2018. The samples in time batches 1–8 were collected and preserved by the South China Sea Fisheries Research Institute (Guangdong, China). Time batch 9 (HS) were oysters purchased in July 2019 from the Huangsha (HS) Aquatic Product Market in Guangzhou, Guangdong Province, but their original farming location was ZH. The samples in that batch were collected and preserved by Guangdong Magigene Biotechnology Co., Ltd (Guangzhou, China). Details on the total samples are given in Table S1.

For time batches 1–6 and 8, the tissues (including gills, mantle, and hepatopancreas) from three adult individuals were mixed to form single samples. For time batch 7, a 1-mL syringe was used to draw hemolymph from the pericardial cavity of the individuals, and then 5–8 individuals were mixed to form single samples. The tissue and hemolymph samples (*n* = 35) were all quickly frozen in liquid nitrogen, temporarily stored with dry ice during transportation, and placed in an ultra-low-temperature freezer at − 80 °C for long-term storage.

### VLP enrichment

All 35 samples were processed to enrich for VLPs as described by Wei et al. [100, 101] and using the online protocols (https://doi.org/10.17504/protocols.io.m4yc8xw). First, 500 mg of mixed tissue (including gills, mantle, and hepatopancreas) was dissected and ground to powder in liquid nitrogen. The powder was further homogenized in approximately 2–5 volumes of sterile SB buffer (0.2 M NaCl, 50 mM Tris–HCl, 5 mM CaCl2, 5 mM MgCl2; pH 7.5). After three rounds of freezing and thawing, the pellets were resuspended entirely in 10 volumes of pre-cooled SB buffer. For the hemolymph sample, 10 mL hemolymph was mixed with an equal volume of 2 × SB buffer and then directly subjected to three rounds of freezing and thawing. The following steps were the same for the tissue and hemolymph samples. All the samples were centrifuged at 1000, 3000, 5000, 8000, 10,000, and 12,000 × *g* for 5 min each at 4 °C using a 3K30 centrifuge (Sigma, Osterode am Harz, Germany), and the supernatants were retained. Cell debris, organelles, and bacterial cells were further removed using a Millex-HV filter with 0.22-μm pore size. The filtrates were transferred to ultracentrifuge tubes containing 28% (w/w) sucrose using a syringe. The tubes were transferred to an ice bath for 10 min before centrifugation in a Himac CP 100WX ultracentrifuge (Hitachi, Tokyo, Japan) at 300,000 × *g* for 2 h. Supernatants were discarded and the precipitates were fully resuspended in 720 μL of water, 90 μL 10 × DNase I Buffer, and 90 μL DNase I (1 U/μL) and then incubated at 37 °C with shaking for 60 min, followed by storage overnight at 4 °C, before being transferred to 2-mL centrifuge tubes.

### Viral nucleic acid extraction and amplification

Total nucleic acid was extracted from the VLPs using an HP Viral DNA/RNA Kit (R6873; Omega Bio-Tek, Norcross, GA, USA); carrier RNA was not used, to avoid potential interference with sequencing results. A Qubit™ dsDNA HS Assay Kit (Q32851) and Qubit™ RNA HS Assay Kit (Q32855) (Thermo Fisher Scientific, Waltham, MA, USA), respectively, were used to quantify the concentrations of dsDNA and RNA.

Virome studies are highly reliant on amplification because the viral biomass in natural samples is very low [4, 71]. Because most available amplification methods introduce bias, it is challenging to study viromic sequencing data quantitatively [23, 68]. Here, a REPLI-g Cell WGA & WTA Kit (150052; Qiagen, Hilden, Germany), which is based on the multiple displacement amplification (MDA) method, was used to uniformly amplify the whole genomes (WGA) and whole transcriptomes (WTA) [35, 67, 70]. MDA has many significant advantages over other amplification methods, such as replicating up to 70 kb, more-even coverage, and 1000-fold higher fidelity than Taq polymerase amplification [35, 87], which make MDA widely used in virome studies.

We used WGA and WTA to construct libraries in four batches of mixed tissues, which accounted for 70% (38/54) of all libraries (Table S1). To better compare the RNA and DNA virus communities, we specifically compared differences in the viral communities obtained with the two amplification methods using the same batches of samples (*n* = 18) (Table S1: Time_Batch_ID #2–4) at the same time. RT-WGA is a modified protocol that simultaneously amplifies DNA and RNA [49, 101]. In this study, 14 libraries were constructed based on RT-WGA, including hemolymph and mixed tissue samples (Table S1). The main reason for using RT-WGA is to simultaneously detect both DNA and RNA potential viral pathogens in diseased batches (Table S1: Time_Batch_ID #6 and #7), for the sake of cost efficiency. The steps for the WGA, WTA, and RT-WGA methods were according to the online protocols (https://doi.org/10.17504/protocols.io.m5vc866). For WTA, there is a “DNA wipeout” step before reverse transcription that aims to remove DNA altogether, but this step is not part of the WGA and RT-WGA protocols. Compared with the protocols of WTA and RT-WGA, the WGA protocol skips the reverse transcription reaction to avoid amplifying RNA in the downstream reaction. In addition, two other samples were directly subjected to random shotgun library preparation using a Nextera XT DNA Library Preparation Kit (Illumina) following the manufacturer’s protocol. Because of the limited data quality and sample number, these two libraries were not included in the following analysis of virus diversity.

### Library construction and sequencing

Amplified DNA was quantified by gel electrophoresis and Nanodrop 2000 spectrophotometer (Thermo Fisher Scientific) and randomly sheared by ultrasound sonication (Covaris M220) to produce fragments of ≤ 800 bp. The sticky ends were repaired, and adapters were added using T4 DNA polymerase (M4211, Promega, USA), Klenow DNA polymerase (KP810250, Epicentre), and T4 polynucleotide kinase (EK0031, Thermo Fisher Scientific, USA). Fragments of 300–800 bp were collected after electrophoresis. After amplification, libraries were pooled and subjected to 150-bp, 250-bp, or 300-bp paired-end sequencing on the NovaSeq 6000, HiSeq X Ten, and MiSeq platforms (Illumina, USA). Considering that the RT-WGA libraries were likely to have higher virus diversity than the WGA and WTA libraries [100], they were sequenced with higher depth and thus produced better assembly results (Table S1).

### Virus detection and quantification based on reference viral genomes

Instead of using the traditional read alignment tools such as BLAST, BWA, and Bowtie2, we used FastViromeExplorer [94], a pipeline developed for fast and accurate virus detection and quantification in metagenomics data. FastViromeExplorer filters the alignment results based on minimal coverage criteria and the minimal number of mapped reads and accurately reports virus types and relative abundances. The program Kallisto v0.43.1, integrated with FastViromeExplorer, was used with the default settings to map clean reads against three reference databases: the National Center for Biotechnology Information (NCBI) Reference Sequence (RefSeq) database, Global Ocean Virome database (GOV) [80], and the Integrated Microbial Genome/Virus (IMG/VR) system, separately, to generate a reference abundance table. The RefSeq database (March 2021 update) contained 14,042 viral genomes or genome segments; GOV [80] included 298,383 epipelagic and mesopelagic viral contigs; and IMG/VR contained 125,842 metagenomic viral contigs of the set of sequences collected from the Joint Genome Institute’s Earth Virome project [66].

### Virus detection and quantification based on de novo assembly (vOTU annotation)

High-quality clean reads were trimmed using fastp v0.20.0 [17] (options: –correction, –trim_poly_g, –trim_poly_x, –overrepresentation_analysis, –trim_front1 = 16, –trim_tail1 = 2, and –length_required = 50), and reads that matched the Illumina sequencing adapters were removed (option: –detect_adapter_for_pe). The clean reads in libraries that were in the same assembly group were pooled and assembled using MEGAHIT v1.2.9 [51] with the default settings. Only contigs longer than 800 bp were kept. To detect low-abundance contigs, clean reads that did not map back to the first round of assembled contigs were reassembled for two additional rounds, and then all remaining reads were pooled and assembled together. Contigs from all four assembly rounds were pooled and clustered at 97% global average nucleotide identity with at least 90% overlap of the shorter contig using cd-hit-est v4.8.1 (options: -aS 0.9 -c 0.97 -G 1 -M 0 -T 0 -g 1) [50], resulting in 3,347,421 nonredundant contigs (Fig. 1A).

The nonredundant contigs were annotated using Diamond v0.9.24.125 (options: -e 1e-10, –max-target-seqs 50) against the NCBI nr database (March 2021 release). Among them, 728,784 (21.77%) of the total contigs were annotated as the viral origin (i.e., vOTUs); 7.68% were Eukaryota, 0.34% were Archeae, 21.59% were bacteria, 0.82% were unclassified cellular organisms, and 47.89% were of unknown origin (Fig. 1A). FastViromeExplorer was used with the default settings to map the clean reads against the vOTU contigs to obtain the vOTU abundance table.

### Viral genome integrity, taxonomy, and auxiliary metabolic gene analysis

The viral genome completeness of assigned contigs was tested using CheckV v0.7.0 and its associated database [59, 60]. After removing false-positive contigs that matched more host genes than viral genes, 3,473 nearly complete viral genomes were obtained.

Three methods (Diamond, vContact2, and PhaGCN) were used to determine the taxonomy of the viral contigs at the family level. Diamond annotations were further processed using two scripts (daa2rma and rma2info) in MEGAN6 [38] with default parameters, and parsed to taxonomy annotations. The advantage of Diamond is that there is no minimum length requirement for query sequences; however, it has three drawbacks: low accuracy, low annotation rates, and inaccurate taxonomy of NCBI. PhaGCN is a novel semi-supervised learning model that combines the strengths of a BLAST-based model and a learning-based model using a knowledge graph [83]. For comparison purposes, only vOTUs of > 10 kb were compared using PhaGCN and vContact2 with default parameters.

To mine the auxiliary metabolic genes (AMGs) from DOV, Vibrant v1.2.1 [45] was used. Salmon v1.5.2 [69] was used with default settings to map clean reads against the AMG dataset to obtain the AMG abundance table.

### Viral contamination assessment

The experimental preparation for viromic sequencing involves the use of various reagents, many of which have been proved to carry contaminated viral sequences of unknown origin [32]. The extent of viral contamination in common laboratory components, especially viruses with small single-stranded DNA (ssDNA) genomes, has been reported previously [3, 72].

To assess the viral contaminant level in this study, all the 3,347,421 nonredundant contigs (≥ 800 bp; not only viral contigs) were used as queries in a BLASTN search (with the parameters set as 95% identity and 95% query coverage) against the approximately 500 contaminant viral sequences reported by Asplund et al. [3] and Porter et al. [72]. We found little evidence of viral contamination, no sequences matched with 100% identity, no expected circoviruses or RNA viruses were detected, and most of the alignments were with dsDNA phages (Additional file 1). The 3473 near-complete viral genomes were used as queries in the same BLASTN search, but no matches were found. We also used Salmon v1.5.2 to map all the clean reads in the DOV libraries to the contaminant viral sequences. The mapping rates for most of these libraries were < 0.01% (Additional file 2), which is consistent with the BLASTN results.

### Viral community and statistical analysis

In this study, the transcripts per million (TPM) value was used to represent the relative abundance of the reference viral genomes, vOTUs, and AMGs. Based on the TPM-transformed abundance table, R and Excel were used to analyze the corresponding viral diversity and community structures. The vegan and ggplots R packages were used to calculate α-diversity indexes and plot the nonmetric multidimensional scaling (NMDS). Analysis of variance (ANOVA) and Tukey’s HSD were used to test the differences between groups, with the significance level set at 0.05. For the Procrustes analysis, the characteristic axis coordinates of NMDS were extracted as the input of the Procrustes function, and the protest function was used to perform the substitution test to evaluate the significance of the results. All the figures in this study were output using basic plotting tools (including R v4.2.1, Gephi v0.9, and iTol v6) and Excel and finally combined and adjusted in Adobe Illustrator CC.

## Results and discussion

### Overview of the Dataset of Oyster Virome (DOV)

For this study, we used 35 samples of mixed tissue or hemolymph from *Crassostrea hongkongensis* collected at nine time points and from seven major oyster farming areas along the south coast of China (Fig. 1; Table S1). Fifty-four oyster virome libraries were constructed using three primary amplification methods (WTA, WGA, and RT-WGA) and then sequenced (Table S1). A total of 3,347,421 nonredundant contigs (of ≥ 800 bp) were obtained after assembly. Among them, 728,784 (21.77%) were annotated as viral origin by comprehensive blast (Fig. 1A), which we called the DOV. The viral contigs were assembled mainly from the RT-WGA libraries of hemolymph samples with higher sequencing coverages (Fig. 1B). Rarefaction curves (Fig. 1C) show that the sequencing depths were valid, and the vOTU numbers in the WTA libraries were the lowest among all the libraries.

Notably, the ratio of viral reads (mapping rate) varied greatly depending on the reference databases that were searched (Fig. 1E). The mapping rate of de novo assembled vOTUs (29.81%) was much higher than the mapping rates of the RefSeq (NCBI viral reference genomes) (3.50%) and the RefSeq plus two other public virus datasets, GOV and IMG/VR (12.06%) (Fig. 1E; Table S1). The higher mapping rates of vOTUs confirmed that the VLP enrichment protocol was effective [53, 101], indicating that filter-feeding oysters can efficiently accumulate environmental viruses. The low mapping rates of the reference genomes (3.50% and 12.06%) imply that the viruses found in the oysters were largely previously unknown. To our knowledge, this is the biggest viral metagenomic dataset currently available for any marine animal.

### Viruses in oysters

Compared with the extensive studies of marine DNA viruses, investigations of oyster-related virus have focused mainly on transcriptomic data and RNA viruses. Rosani et al. [75–77] assembled 26 novel and nearly complete RNA virus genomes from the public transcriptomic data of *C. gigas* and *C. corteziensis*, and Zhang et al. [107] reported four new RNA virus genomic fragments from *C. gigas*, which were recovered from a virome survey of marine invertebrates. Another 33 novel RNA viruses were identified from mixed bivalve samples (including two oyster species, *C. hongkongensis* and *C. ariakensis*) [85]. To explore RNA viruses, 33 related libraries (including 19 WTA and 14 RT-WGA libraries) were constructed in this study (Table S1). However, we only recovered 4,958 RNA vOTUs, which accounted for 0.68% of all the viruses in the DOV, and all of them were classified as unknown Riboviria (Fig. S1). Compared with the substantial DNA virus sequence database, the dataset of RNA viruses is exceptionally small. Recently, new approaches were used to optimize the discovery methods of RNA viruses, which has greatly expanded the available RNA virus catalog [105, 62, 103]. We anticipate that more RNA viruses associated with oysters will be explored if these new approaches and the expanded dataset are used.

**Fig. 2:**
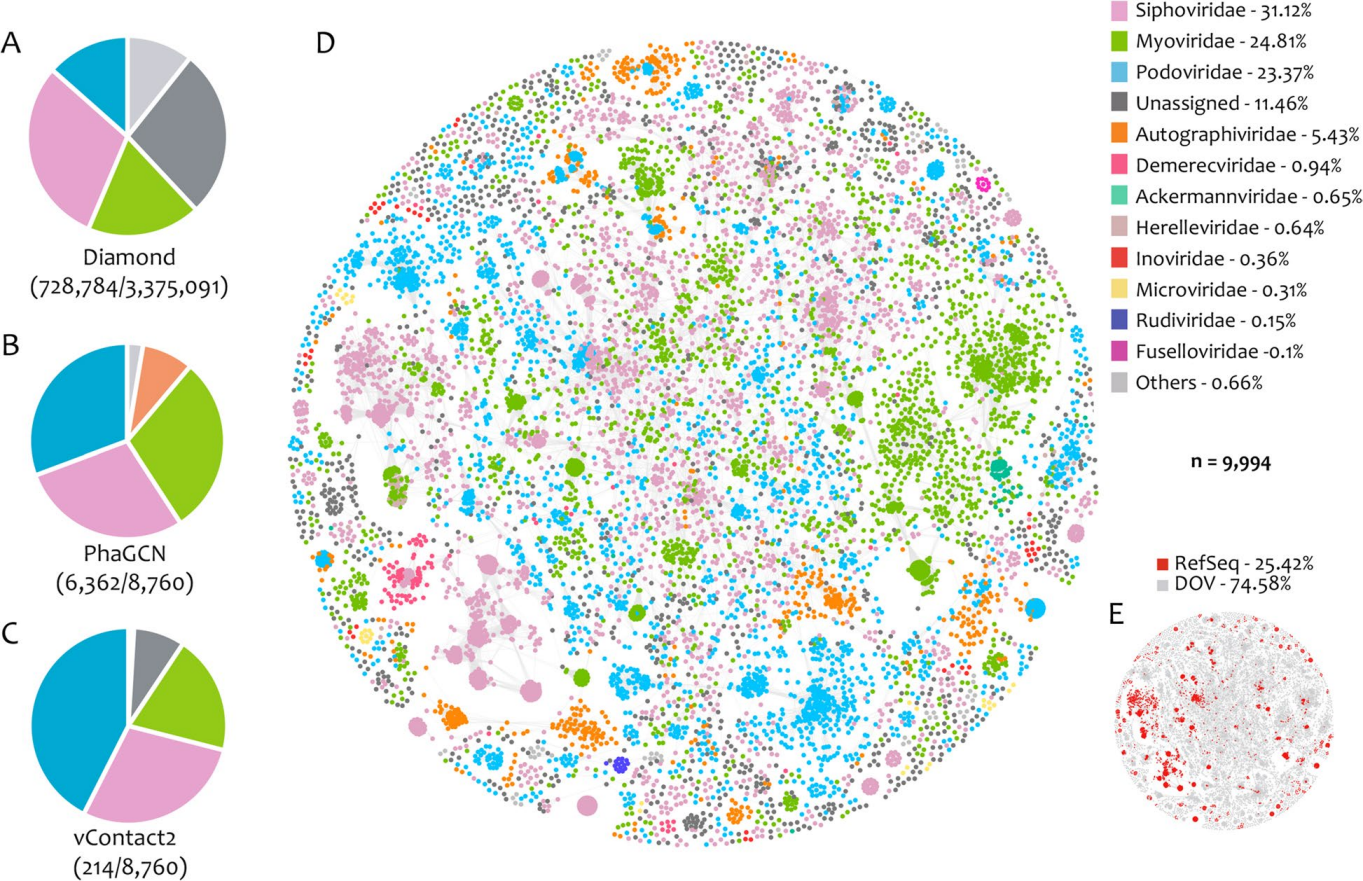
Taxonomy classification of the Dataset of Oyster Virome (DOV) at the family level. **A**–**C** Pie charts showing the proportion of different viral families in the total viral contigs (vOTUs) longer than 800 bp. The vOTUs were classified using Diamond v0.9.24.125 (**A**), and vOTUs longer than 10 kb were classified using PhaGCN (**B**) and vContact2 (**C**). The numbers in parentheses indicate the number of vOTU successfully classified/total number of queries. **D**, **E** vContact2 networks constructed with vOTUs and NCBI RefSeq viral genomes (release March 2021) longer than 10 kb showing they have the same topology. The colors of the nodes indicate different PhaGCN families (**D**), and their sources (**E**). *n*, total number of nodes in vContact2 networks. The percentage of each family or source in **D** and **E** is listed after the corresponding legends

Ostreid herpesvirus is the most extensively studied DNA viral pathogen for oysters and many other aquaculture bivalves [18, 24, 28, 74, 77]. Compared with RNA viruses in the DOV, which have lower diversity, large numbers of DNA viruses were found to have dominated at all the sampling sites (Fig. 1), which indicates the importance of DNA viruses in oysters and the marine environment. Consistent with the results of Dupont et al. [22], viruses in the order Caudovirales dominated the oyster virome (Figs. 2 and S1), just as they dominate the public dataset and culture collections [44]. The top-three Caudovirales families in the DOV were Siphoviridae (28.5–30.61%), Podoviridae (13.46–42.52%), and Myoviridae (18.36–29.61%) (Fig. 2A–C). Considering the primary bias of MDA on circular ssDNA genome, Microviridae and Circoviridae accounted for only 2.23% of all the viruses (Fig. S1), which means their diversity may be less than 2.23% and much lower than the diversity of the dsDNA viruses in the DOV.

BLAST-based taxonomy of short contigs has limited accuracy [41] and a large proportion of them (> 30%) could not be assigned at the family level (Fig. S1). In view of this, PhaGCN was used and successfully classified 6,362 out of 8,760 large vOTUs (of ≥ 10 kb) (Fig. 2B), which exceeded the number classified by vContact2 (214/8,760) (Fig. 2C), and the percentage of unassigned vOTUs decreased to 11.46% (Fig. 2D). Impressively, the DOV nodes (vOTUs) accounted for 74.58% of the total nodes, whereas the RefSeq nodes account for only 25.42% in the vConTACT2 network (Fig. 2E), indicating that current knowledge about the ocean virosphere is far from sufficient.

### Near-complete viral genomes.

**Fig. 3:**
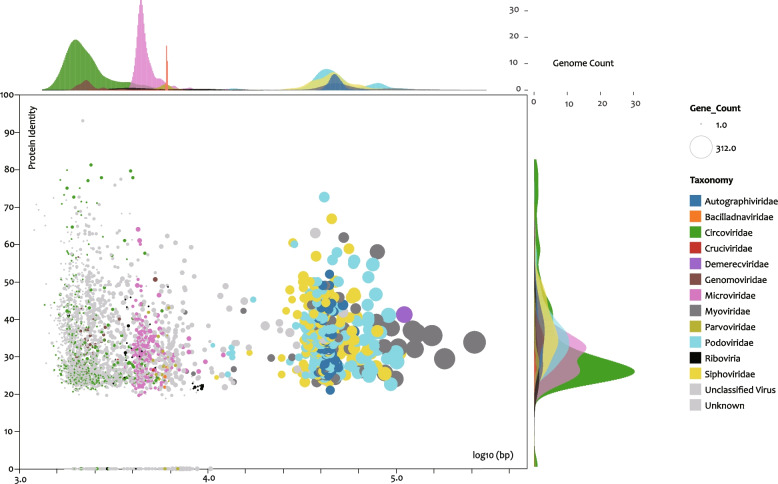
Scatter plot of complete and near-complete viral genomes in the Dataset of Oyster Virome (DOV). *X*-axis, log10 value of viral genomic length; *Y*-axis, protein identity between DOV genomes and CheckV reference genomes. The diameter of the circles indicates the gene count in the genomes. Colors indicate different viral families obtained by combining the PhaGCN and Diamond v0.9.24.125 results. The density histograms parallel to the *X*- and *Y*-axes show the distribution of the genome count for the corresponding virus families

A total of 3,473 viral contigs with > 90% genomic completeness (including 27 RNA viral genomes) were identified (Figs. 3 and S2; Table S2). The genome lengths were 1,206–60,277 bp, and the GC content was 24.74–65.70% (Fig. S2). The encoded proteins shared a maximal identity of 0–93.10% (but mainly in the range of 20–40%) with known viral proteins (Fig. 3; Table S2), which again indicated that most of the genomes represented new viral categories. Only 16 of them clustered with nonredundant reference genomes of CheckV, with 95% average nucleotide identity and 70% alignment fraction of contigs. We considered both unknown and unclassified sequences (gray dots in Fig. 3) as representing novel viruses at the family level, which account for 67.1% (2,330) of the total (Table S2). The classified genomes belonged to at least 11 DNA virus families; viruses in the order Caudovirales included the Podoviridae (173), Sipoviridae (136), Myoviridae (66), and Autographiviridae (46) (Fig. S2). Circoviridae (order Cirlivirales) and Microviridae (order Petitvirales) were the most abundant families, accounting for 11.27% (396) and 6.98% (240) of the classified genomes, respectively (Fig. 3; Table S2).

Among the viruses recognized at the family level, the “Cruciviridae” clade, Genomoviridae, Parvoviridae, and Circoviridae have the potential to infect animals or even humans. The red fire ant is the only known host of members of the Cruciviridae. This species may be related to some small arthropods that are symbiotic or filter-fed in oysters. Viruses in the family Genomoviridae have been recorded to be hosted by a wide range of animals, such as humans [98], the capybara [26], tortoises [15], birds [97], and many other terrestrial animals. Hosts that have been identified to be infected by members of the Parvoviridae include sea stars [39], species of *Crassostrea* [43] and *Fenneropenaeus* [8], seals [9], humans [21], and pigeons [40]. In addition to the well-known circovirus hosts, namely pigs [93] and birds [102], circovirus has also been found in several fish species [20, 57, 58], gulls [95], whales [47], and humans [86]. Notably, the discovery of a variety of potential avian viruses reminds us that water contamination from bird feces may be a potential source of marine viruses,therefore, oysters may play an important role as repositories and transmission hotspots of these viruses.

### Oyster-related circoviruses

Circovirus was first described in pigs [93], and together with Cyclovirus, which is found in numerous animal hosts, it forms the family Circoviridae [6]. Circoviruses are among the smallest animal viruses with an unenveloped icosahedral structure (12–27 nm in diameter), with genomes that mainly include two genes that encode replication initiator protein (Rep) and capsid protein. Circovirus-like genomes have been commonly uncovered in some virome studies, especially investigations employing the MDA method. However, most of the samples analyzed in these studies were environmental or fecal samples [14, 19, 108], which means that it is difficult to determine the exact host of those circovirus-like sequences. As shown from the viral proteomic tree (Fig. S2), circovirus-related branches were widely dispersed and mixed with unannotated branches, implying that many putative circovirus clades are yet to be identified. The fact that all currently known hosts of circoviruses are in clade Bilateria of kingdom Animalia (Virus-Host Database, May 2021: https://www.genome.jp/virushostdb) suggests that the circoviruses in the DOV were most likely ones hosted by oysters or other multicellular organisms associate with oysters. Although genetic variation in circovirus can occur fast, similar to the properties of some RNA viruses [25], finding so many circovirus-like genomes in one animal species was quite unexpected.

Furthermore, we used the Rep sequences of circoviruses recorded by the International Committee on Taxonomy of Viruses (ICTV) as queries and mined out 1390 and 8763 nearly complete circovirus-related Rep sequences from the NCBI nr and DOV, respectively, by iterative BlastP searches. Similarity clustering of the identified Rep sequences (Fig. S3) shows that the circovirus-related sequences are very diverse. With the exception of the two Circoviridae genera, Circovirus and Cyclovirus, which have been recorded by the ICTV, most of the other clusters contain sequences that have not been clearly classified (Fig. S3). Among them, the sequences from the DOV accounted for 86.3% (6.3 times the percentage from the NCBI nr) and were widely distributed and present in all the clusters. Some clusters even contained only sequences recorded in the DOV, which indicates that the sequences had not yet been discovered (Fig. S3).

**Fig. 4:**
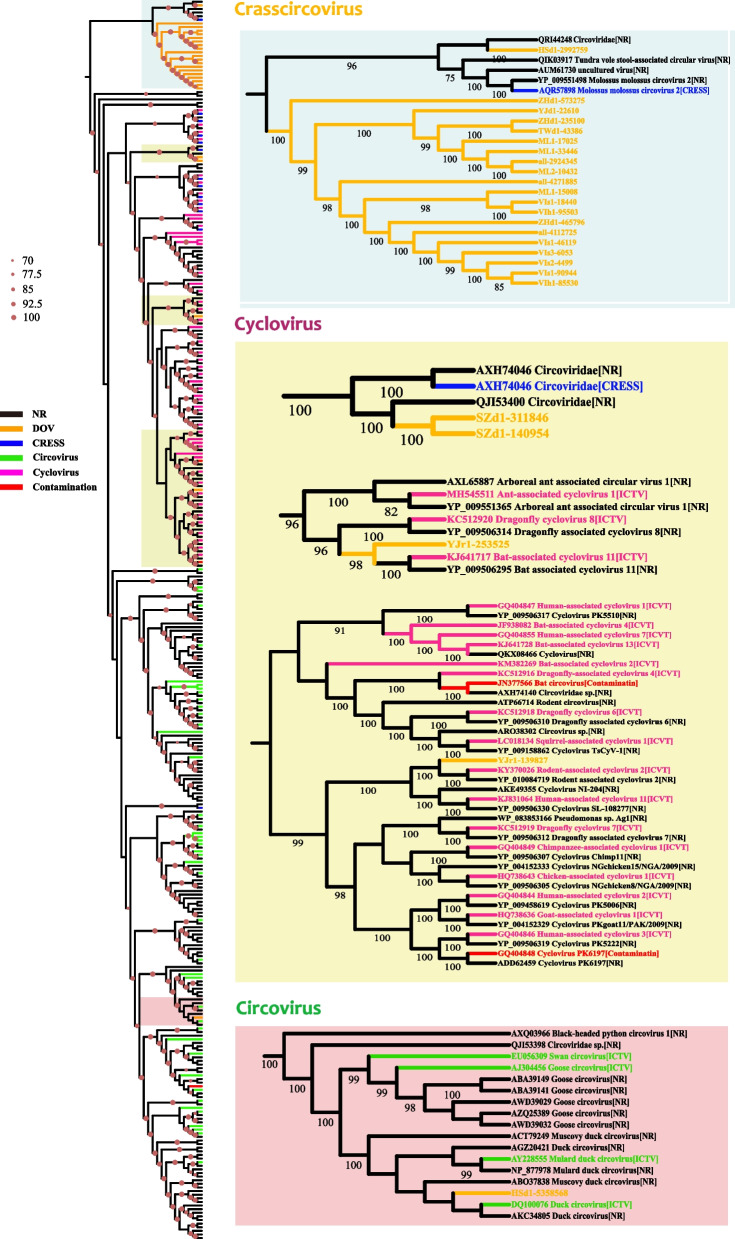
Phylogeny of replication initiator proteins of oyster-related circoviruses. The large tree on the left shows the phylogeny of all proteins in the clusters of the two standard Circoviridae genera recorded by the International Committee on Taxonomy of Viruses (ICTV) (Fig. S3), namely Cyclovirus (green) and Circovirus (violet). The diameter of red dots on branches represents the bootstrap value, and only values above 70 are shown. Small trees on the right are enlarged branches from the tree on the left. Background colors indicate different viral genera: light blue, Crasscircovirus; yellow, Cyclovirus; pink, Circovirus. Colors of branches indicate data origins: orange, Dataset of Oyster Virome (DOV); gray, NCBI nr database; blue, CRESS viruses from Porter et al. [72], green, circoviruses from the ICTV; violet, cycloviruses from the ICTV; red, contaminant sequences from Asplund et al. [3] and Porter et al. [72], black, other NCBI nr sequences

We also constructed a phylogeny (Fig. 4) using the Rep sequences that clustered with the circoviruses and cycloviruses (Fig. S3). The results showed that most of the Rep sequences from the DOV were on an independent branch separate from the Circovirus and Cyclovirus branches and distant from the branches of contaminant sequences (excluding the possibility of reagent contamination). We considered that these Rep sequences from the DOV represented a new oyster- or bivalve-specific genus under Circoviridae, and we tentatively named it Crasscircovirus (Fig. 4). Five of the DOV sequences were scattered in different Circovirus and Cyclovirus branches (Fig. 4). These findings suggest that oysters (and possibly bivalves) may be hotspots of circoviruses. Whether these circoviruses are pathogens or live as symbionts in oyster hosts and whether they will spill over to other marine animals, similar to the behavior of coronavirus in bats, are topics that merit further study [96].

### RNA viruses versus DNA viruses

Most previous virome studies focused only on DNA or RNA viruses. Quantitatively comparing the diversity and abundance among RNA and DNA viruses in real environments will likely be very interesting [34, 89, 109]. However, so that we could compare the results, in this study, we used various targeted amplifications to compare DNA and RNA viruses in the same sample separately (WGA and WTA) or simultaneously (RT-WGA).

First, our study shows different amplification strategies can efficiently target different genomes, because the vOTUs of RNA viruses in the WTA libraries significantly outnumber those in the WGA libraries, and vice versa for the DNA viruses (Fig. S4A, B). Second, although the differences were not significant, the α-diversity of WGA libraries seems to be higher than WTA libraries (Fig. S4D–F), which is consistent with previous observations (Figs. 1C and S1). It seems to be common that the diversity of DNA viruses in nature and public databases is higher than the diversity of RNA viruses [48, 78, 79]. However, further studies are needed to confirm the conclusion that DNA viruses are more diverse than RNA viruses. Furthermore, the extremely high mutation rates of RNA genomes challenged their detection recall of alignment-based annotations [33, 85], and the instability of RNA genomes and potential amplification bias also complicated the comparisons.

Notably, although the diversity of the RNA viruses detected seemed low, their abundance (viral reads ratio) in the WTA libraries was similar to that in WGA libraries and significantly higher than found in RT-WGA libraries (Fig. S5A). However, because the samples and tissues used by RT-WGA differed from those used by WTA and WGA, we are unable to determine why the RT-WGA libraries showed a relatively low viral reads ratio. Interestingly, the ratio of Riboviria reach up to 70% (Table S1; Fig. S5B), when only a tiny ratio of DNA virus transcripts was detected in some WTA libraries (i.e., ChSZ1604Ra and ChSZ1604Rb) (Fig. S5C). The detection of transcripts of DNA viruses in the RNA libraries probably indicates that these DNA viruses are actively replicating in the host cells. However, it does not prove that they are pathogens in oysters, because they could be the viruses of other symbiotic organisms. Nonetheless, the WTA libraries that contained an ultra-high proportion of RNA viruses merit further investigation to determine which kinds of RNA viruses are dominant in the samples and to understand why RNA and DNA viruses seem to utilize different replicating and ecological lifestyles.

### Viral communities

MDA introduces bias by prioritizing circular ssDNA genome [7], and this may have led to the > 80% abundance of circular ssDNA virus in several libraries in this study (Fig. S5C). Parras-Moltó et al. [68] found that ordination plots based on dissimilarities among vOTU profiles showed perfect overlapping of related amplified and unamplified viromes and strong separation from unrelated viromes, which showed that MDA can be used for virus community studies. Studies of virus communities can help determine whether the viruses enriched in oysters can be regarded as an organic whole, similar to viruses in the marine environment, or are simply a random and incidental assembly, as well as whether the community can respond to external influences.

**Fig. 5:**
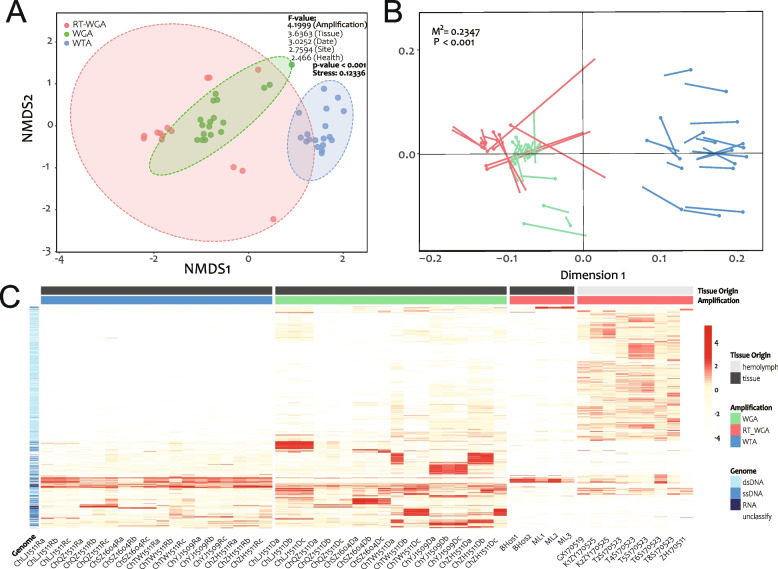
Viral community in the Dataset of Oyster Virome (DOV). **A** Nonmetric multidimensional scaling (NMDS) analysis shows the clusters of DOV libraries according to amplification groups. Nonparametric multivariate analysis of variance (permanova) was used. RT-WGA, reverse transcription and whole genome amplification; WGA, whole genome amplification; WTA whole transcriptome amplification. **B** Procrustes analysis of NMDS coordinates of viral communities based on comparisons of reference genomes (RefSeq, GOV, and IMG/VR) and de novo assembled viral contigs (vOTUs). **C** Heatmap of DOV vOTUs. The vOTUs clustered by the Euclidean method and colored by the viral genome types (dsDNA, ssDNA, RNA, and unclassified) are shown on the *Y*-axis. The DOV libraries ordered by amplification strategy (WGA, RT_WGA, and WTA) and tissue origin (hemolymph and mixed tissue) are shown on the *X*-axis

We first evaluated the correlation among various community parameters, including the vOTU counts, the ratio of viral reads, variation in the diversity indexes, and the quantity and quality of sequencing reads (Fig. S6). The α-diversities correlated well among three approaches to deciphering communities (based on the RefSeq, vOTU, and AMG datasets) (Fig. S6), which indicates that the methodologies we used for community analysis verified each other. Second, as we expected, targeted amplification plays a decisive role in the virus community (Fig. 5A), and this was further verified by our determination of the communities based on reference genomes (Fig. 5B). Besides the amplification method, the obviously different virus abundance patterns, as revealed by the heatmap (Fig. 5C) and the *F*-value ranks (Fig. 5A), showed prominent differences between tissue groups. Even in a semi-open circulatory system, the virus community in the tissue submerged by hemolymph was quite different from that in the hemolymph itself, which shows that different host tissues had a selective effect on the viruses.

Importantly, although the influence of health status, sampling site, and sampling time on the whole community did not seem to be significant (low *F*-value) (Fig. 5A), we still found significant differences in both the α- and β-diversity (NMDS) between all healthy and diseased samples (Fig. S7A, C). The α-diversity of moribund groups was relatively high, perhaps signaling that the decrease in immunity caused by disease led to an increase of opportunistic pathogens and their bacteriophages in the host. Dupont et al. [22] found that the pathogen OsHV-1 μVar virus dominated the hemolymph virome of *C. gigas* during a disease outbreak, further leading to lower viral diversity than detected in healthy controls. However, the expected differences between moribund and healthy groups were not detected in the parallel cohorts in this study (Fig. S9B, C), which suggested that the virus may not be the oyster pathogen.

Geographic origin (sampling site) also substantially influenced the community. Samples from the same location tended to aggregate, and significant differences in α-diversity were observed from the WGA and WTA groups separately (Fig. S8). The influence of the habitat on the microbiome of the host has been reported in many animals [29, 46, 81, 90] and environmental variations may be one reason for the differences [64]. However, unlike freely swimming fish, oysters are sedentary and filter large volumes of the surrounding water daily [5, 65]. The influence of site on the viromics (viral community) was weaker than that of the time point (lower *F*-value) (Fig. 5A), and this was also reflected in the proportion of unique vOTUs (i.e., those detected in only one group) (Fig. S9). The relatively high proportion of unique vOTUs in the time-batch groups implies that viral communities are dynamic with time, and the low proportion of unique vOTUs between sites indicates that viruses were actively exchanged among locations. However, because of the limited sample number, these results need further verification.

### Auxiliary metabolic genes (AMGs)

Viruses play essential roles in metabolic regulation in the marine ecosystem [10, 11, 91]. Like marine viruses, a large number (9,091) of AMGs were identified from the DOV. They were assigned to 12 KEGG (Kyoto Encyclopedia of Genes and Genomes) metabolism categories and 98 pathways (Table S3). Among them, pathways associated with the metabolism of cofactors and vitamins, amino acids, energy, and carbohydrates were significantly enriched (Fig. S10A), which is similar to the results obtained for other marine viromes [16, 36, 37]. Importantly, the AMG community (Fig. S10B) showed consistency with the vOTU community (Fig. S10C), and the richness and Shannon index showed positive correlations between the two communities (Figs. S6, S10D, E). These findings indicate that the oyster viromic function was closely related to that of the species community. Although it is difficult to know which of these is the cause and which is the result, this discovery provides clues that can improve our understanding of the ecological function of the virome in oysters. In addition, the previous finding that viruses with large genomes tend to encode more AMGs than viruses with small genomes, and that they provide ecological functions beyond sustaining basic infection and proliferation [42], is supported by the results presented in Fig. S10F.

## Conclusions

Here, we report a comprehensive Dataset of Oyster Virome (DOV) with high resolution, which provides a new resource for studying and understanding the marine virome. Our study describes feasible and targeted protocols for the comparative study of DNA and RNA viromes and suggests that hemolymph may be a suitable tissue for the discovery of viruses in bivalves. Notably, multiple aspects of the research output, including reads recruitment, vOTUs, high-quality virus genomes, and circovirus-related Rep proteins, show that oysters undoubtedly harbor a large, diverse, and unique array of viruses. Oysters may be considered as repositories and transmission hotspots of marine viruses, which is likely an outcome of their filter-feeding lifestyle and the high density of natural populations. In addition, the viral communities in oysters appear to be not random but well organized, and able to respond to changes in host tissues and health state, and in the external environment at both compositional and functional levels. Further studies on the viral community structure and function of bivalves will greatly contribute to the knowledge of their role in coastal microbiome regulation, disease transmission, and potential for protecting and restoring coastal ecosystems.

## Supplementary Information

**Additional file 1:**

**Additional file 2:**

**Additional file 3: Figure S1.** Doughnut chart of the taxonomy classification of all the viral contigs (vOTUs) in the Dataset of Oyster Virome (DOV). The proportion of different viral families and unclassified vOTUs (≥800 bp) in DOV are based on BLAST searches of the results of Diamond v0.9.24.125 against the NCBI nonredundant protein sequence (nr) database (release March 2021).

**Additional file 4: Figure S2.** Viral proteomic phylogenetic tree of complete and near-complete viral genomes in the Dataset of Oyster Virome (DOV). The viral genomes were clustered based on their mutual amino acid identity using ViPTreeGen v1.1.2. The layers from inside to outside show (1) the warning message of CheckV, (2) GC content of the viral genomes, (3) CheckV evaluation methods of genome completeness, (4) log10 value of genomic length, (5) percentage of genome completeness evaluated by CheckV, (6) viral families in order Caudovirales predicted by PhaGCN, and (7) viral families and non-viral annotations of all the genomes obtained by BLAST searches of the results from Diamond v0.9.24.125 against the NCBI nonredundant protein sequence (nr) database (release Mar 2021).

**Additional file 5: Figure S3.** Similarity clustering of circovirus-related replicase proteins in Dataset of Oyster Virome (DOV) and NCBI nr. Dots represent different replicase sequences (n=4,716). Edges represent the score value of the Diamond BlastP results; only scores higher than 185.0 are shown. Network clustering was performed using Gephi v0.9.2 under the Fruchterman-Reingold model. Colors of dots indicate different data origins: orange, Dataset of Oyster Virome (DOV); blue, CRESS from Ashleigh et al. (2021); green, circoviruses from the International Committee on Taxonomy of Viruses (ICTV); violet, cycloviruses from the ICTV; red, contaminant sequences from Asplund et al. (2019) and Porter et al. (2021); grey, other NCBI nr sequences.

**Additional file 6: Figure S4.** Preference of amplification strategies for the viral community and genome types. (A) Counts of RNA, (B) DNA, and (C) unclassified viral contigs (vOTU) using the WGA and WTA strategies. (D) Richness, (E) Shannon, and (F) Simpson indexes of the three amplification strategies: RT-WGA, reverse transcription and whole genome amplification; WGA, whole genome amplification; WTA whole transcriptome amplification. Different lowercase letters indicate significant differences (*P* <0.05; one-way ANOVAs and Tukey-Kramer post hoc comparisons).

**Additional file 7: Figure S5.** Actual and relative abundances of virus taxons in the Dataset of Oyster Virome (DOV) libraries. (A) Comparison of the viral reads ratio among three amplification groups; (B) viral reads ratio and (C) relative abundance of the taxons in the 54 DOV libraries (X-axis). Annotations are based on BLAST searches of the results of Diamond v0.9.24.125 against the NCBI nonredundant protein sequence (nr) database (release March 2021). To facilitate the display, the classifications were not unified at the same taxonomic levels.

**Additional file 8: Figure S6.** Correlation matrix of oyster viral communities. Red labels (n=10), diversity indexes, viral reads ratio, and vOTU counts based on vOTUs mapping results; black labels (n=7), quality related parameters of library construction and sequencing; blue labels (n=4), diversity indexes and viral ratio based on the reference genomes (RefSeq, GOV, and IMG/VR) mapping results; green labels (n=3): diversity indexes based on the auxiliary metabolic genes (AMGs) mapping results.

**Additional file 9: Figure S7.** Influences of health status on the viral community in the Dataset of Oyster Virome (DOV). (A) Nonmetric multidimensional scaling (NMDS) plots of all the libraries (n=54) and (B) the seventh batch (May 2017) (n=9). (C) Comparison α-diversities (Richness, Shannon and Simpson indexes) between healthy and moribund samples corresponding to the NMDS plots in (A) and (B). Blue bar, healthy group; purple bar, moribund group. Different lowercase letters indicate significant differences (P <0.05; one-way ANOVAs and Tukey-Kramer post hoc comparisons).

**Additional file 10: Figure S8.** Influences of sampling sites on the viral community in the Dataset of Oyster Virome (DOV). (A, B) Nonmetric multidimensional scaling plots of different sampling sites of the WTA (A) and WGA (B) groups. (C–F) Comparisons of alpha diversity indexes among sampling sites of the WTA (C. D) and WGA (E, F) groups. The colors are used consistently in the figure. Different lowercase letters indicate significant differences (P <0.05; one-way ANOVAs and Tukey-Kramer post hoc comparisons). WGA, whole genome amplification; WTA whole transcriptome amplification.

**Additional file 11: Figure S9.** Percentage of unique viral contigs (vOTUs) (detected in only one group) under different grouping methods.

**Additional file 12: Figure S10.** Auxiliary metabolic gene (AMG) diversity in the Dataset of Oyster Virome (DOV). (A) Number of detected AMGs assigned to different KEGG metabolic pathways. (B) Nonmetric multidimensional scaling (NMDS) plot of AMG diversity in the DOV libraries (n = 54). (C) Procrustes analysis of NMDS coordinates between the viral contig (vOTU) and AMG communities. The colors are used consistently in (B) and (C): green, WGA; blue. WTA, red. RT-WGA libraries. RT-WGA, reverse transcription and whole genome amplification; WGA, whole genome amplification; WTA whole transcriptome amplification. (D, E) Correlations and linear correlation curves of the Richness (D) and Shannon (E) indexes between vOTUs and AMGs. (F) Correlation between AMG and open reading frame (ORF) counts on the same vOTU.

**Additional file 13 Table S1.** Detailed library grouping information and corresponding metadata.

**Additional file 14: Table S2.** Near-complete viral genomes in the Dataset of Oyster Virome (DOV) identified by CheckV.

**Additional file 15: Table S3.** Counts of auxiliary metabolic genes (AMGs) and corresponding KEGG categories.

## Data Availability

The data set supporting the results of this article has been deposited in the Genome Sequence Archive and Genome Warehouse in National Genomics Data Center (NGDC) under BioProject accession code PRJCA007058 [https://ngdc.cncb.ac.cn/bioproject/browse/PRJCA007058].
