# Correction: A remarkably diverse and well-organized virus community in a filter-feeding oyster

**DOI:** 10.1186/s40168-023-01487-0

**Published:** 2023-02-09

**Authors:** Jing-Zhe Jiang, Yi-Fei Fang, Hong-Ying Wei, Peng Zhu, Min Liu, Wen-Guang Yuan, Li-Ling Yang, Ying-Xiang Guo, Tao Jin, Mang Shi, Tuo Yao, Jie Lu, Ling-Tong Ye, Shao-Kun Shi, Meng Wang, Ming Duan, Dian-Chang Zhang

**Affiliations:** 1 Key Laboratory of South China Sea Fishery Resources Exploitation and Utilization, Ministry of Agriculture and Rural Affairs, South China Sea Fisheries Research Institute, Chinese Academy of Fishery Sciences, Guangzhou, 510300 Guangdong China; 2 College of Fisheries and Life Science, Shanghai Ocean University, Shanghai, 201306 China; 3 Guangdong Province Key Laboratory for Biotechnology Drug Candidates, School of Biosciences and Biopharmaceutics, Guangdong Pharmaceutical University, Guangzhou, 510006 Guangdong China; 4 Tianjin Agricultural University, Tianjin, 300384 China; 5 Present Address: Shanghai Majorbio Bio-Pharm Technology Co Ltd, Shanghai, 201203 China; 6 Guangdong Magigene Biotechnology Co Ltd, Guangzhou, 510000 Guangdong China; 7 School of Medicine, Sun Yat-Sen University, Shenzhen, 518107 Guangdong China; 8 Shenzhen Fisheries Development Research Center, Shenzhen, 518067 Guangdong China; 9 Bureau of Agriculture and Rural Affairs of Conghua District, Guangzhou, 510925 Guangdong China; 10 State Key Laboratory of Freshwater Ecology and Biotechnology, Institute of Hydrobiology, Chinese Academy of Sciences, Wuhan, 430072 Hubei China

**Correction: Microbiome 11, 2 (2023)**

**https://doi.org/10.1186/s40168-022-01431-8**

Following publication of the original article [1], the author reported that there was an error in the affiliations of the authors.

The affiliations of Yi-Fei Fang and Ming Duan were mislabeled.

The affiliation 5 of Yi-Fei Fang should be "Present address: Shanghai Majorbio Bio-Pharm Technology Co Ltd, Shanghai 201203, China".

The affiliation 10 of Ming Duan should be "State Key Laboratory of Freshwater Ecology and Biotechnology, Institute of Hydrobiology, Chinese Academy of Sciences, Wuhan 430072, Hubei, China."

The "Present address" at the beginning of affiliation 6 should be also deleted.

Other authors' affiliations remain unchanged.

The original article has been updated.
