# Supplementary material for: A remarkably diverse and well-organized virus community in a filter-feeding oyster
Source: Microbiome. 2023 Jan 7;11:2. doi: 10.1186/s40168-022-01431-8 (PMC9825006; doi:10.1186/s40168-022-01431-8)
Supplement: Supplementary file 4 — Additional file 3: Figure S1. Doughnut chart of the taxonomy classification of all the viral contigs (vOTUs) in the Dataset of Oyster Virome (DOV). The proportion of different viral families and unclassified vOTUs (≥800 bp) in DOV are based on BLAST searches of the results of Diamond v0.9.24.125 against the NCBI nonredundant protein sequence (nr) database (release March 2021). [file 40168_2022_1431_MOESM3_ESM.pdf]

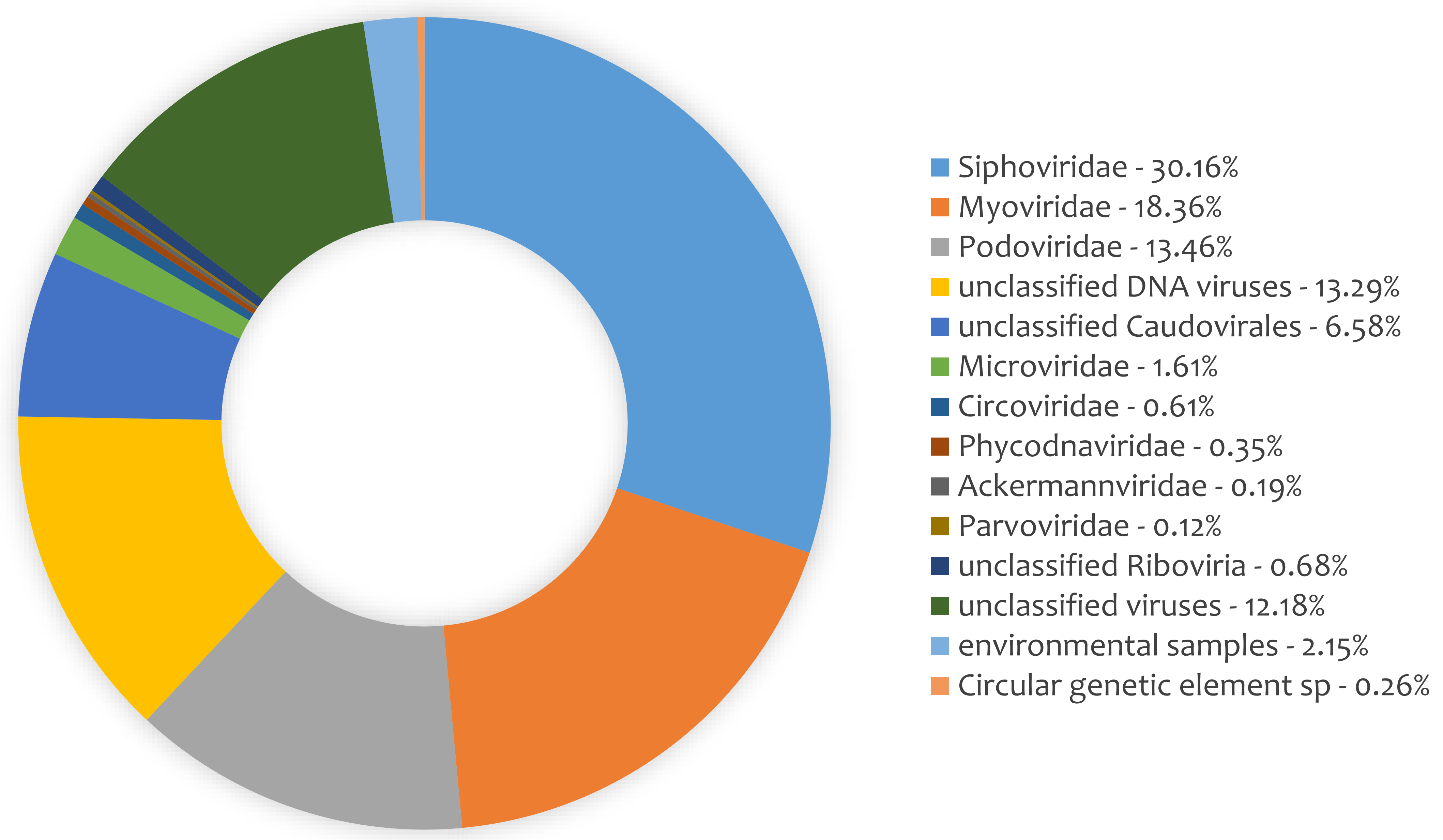
Siphoviridae - 30.16%
Myoviridae - 18.36%
Podoviridae - 13.46%
unclassified DNA viruses - 13.29%
unclassified Caudovirales - 6.58%
Microviridae - 1.61%
Circoviridae - 0.61%
Phycodnaviridae - 0.35%
Ackermannviridae - 0.19%
Parvoviridae - 0.12%
unclassified Riboviria - 0.68%
unclassified viruses - 12.18%
environmental samples - 2.15%
Circular genetic element sp - 0.26%
