# Supplementary material for: A remarkably diverse and well-organized virus community in a filter-feeding oyster
Source: Microbiome. 2023 Jan 7;11:2. doi: 10.1186/s40168-022-01431-8 (PMC9825006; doi:10.1186/s40168-022-01431-8)
Supplement: Supplementary file 5 — Additional file 4: Figure S2. Viral proteomic phylogenetic tree of complete and near-complete viral genomes in the Dataset of Oyster Virome (DOV). The viral genomes were clustered based on their mutual amino acid identity using ViPTreeGen v1.1.2. The layers from inside to outside show (1) the warning message of CheckV, (2) GC content of the viral genomes, (3) CheckV evaluation methods of genome completeness, (4) log10 value of genomic length, (5) percentage of genome completeness evaluated by CheckV, (6) viral families in order Caudovirales predicted by PhaGCN, and (7) viral families and non-viral annotations of all the genomes obtained by BLAST searches of the results from Diamond v0.9.24.125 against the NCBI nonredundant protein sequence (nr) database (release Mar 2021). [file 40168_2022_1431_MOESM4_ESM.pdf]

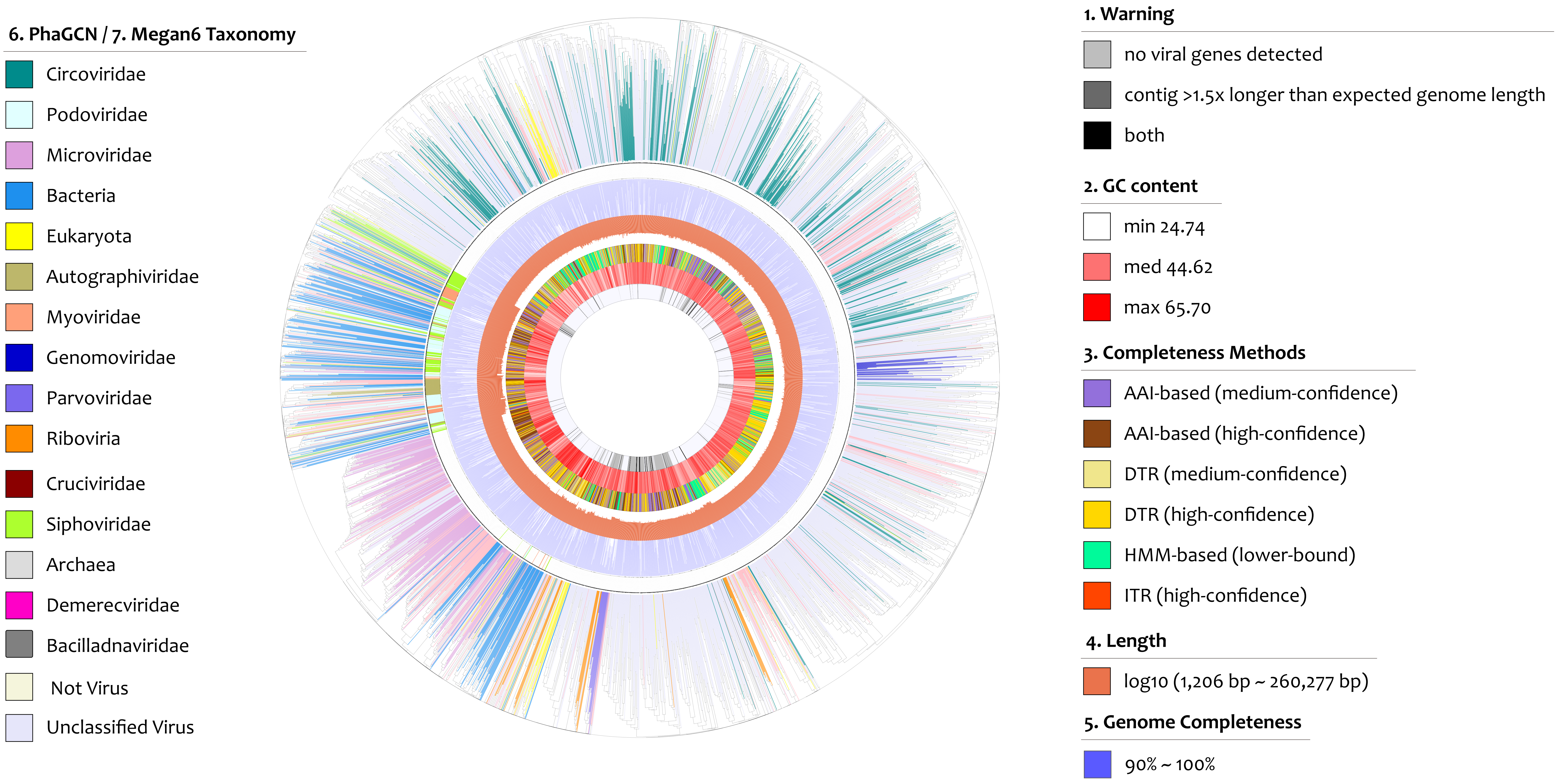
6. PhaGCN / 7. Megan6 Taxonomy
Circoviridae
Podoviridae
Microviridae
Bacteria
Eukaryota
Autographiviridae
Myoviridae
Genomoviridae
Parvoviridae
Riboviria
Cruciviridae
Siphoviridae
Archaea
Demerecviridae
Bacilladnaviridae
Not Virus
Unclassified Virus
1. Warning
no viral genes detected
contig >1.5x longer than expected genome length
both
2. GC content
min 24.74
med 44.62
max 65.70
3. Completeness Methods
AAI-based (medium-confidence)
AAI-based (high-confidence)
DTR (medium-confidence)
DTR (high-confidence)
HMM-based (lower-bound)
ITR (high-confidence)
4. Length
log10 (1,206 bp ~ 260,277 bp)
5. Genome Completeness
90% ~ 100%
