# Supplementary figures and images for: A remarkably diverse and well-organized virus community in a filter-feeding oyster
Source: Microbiome. 2023 Jan 7;11:2. doi: 10.1186/s40168-022-01431-8 (PMC9825006; doi:10.1186/s40168-022-01431-8)

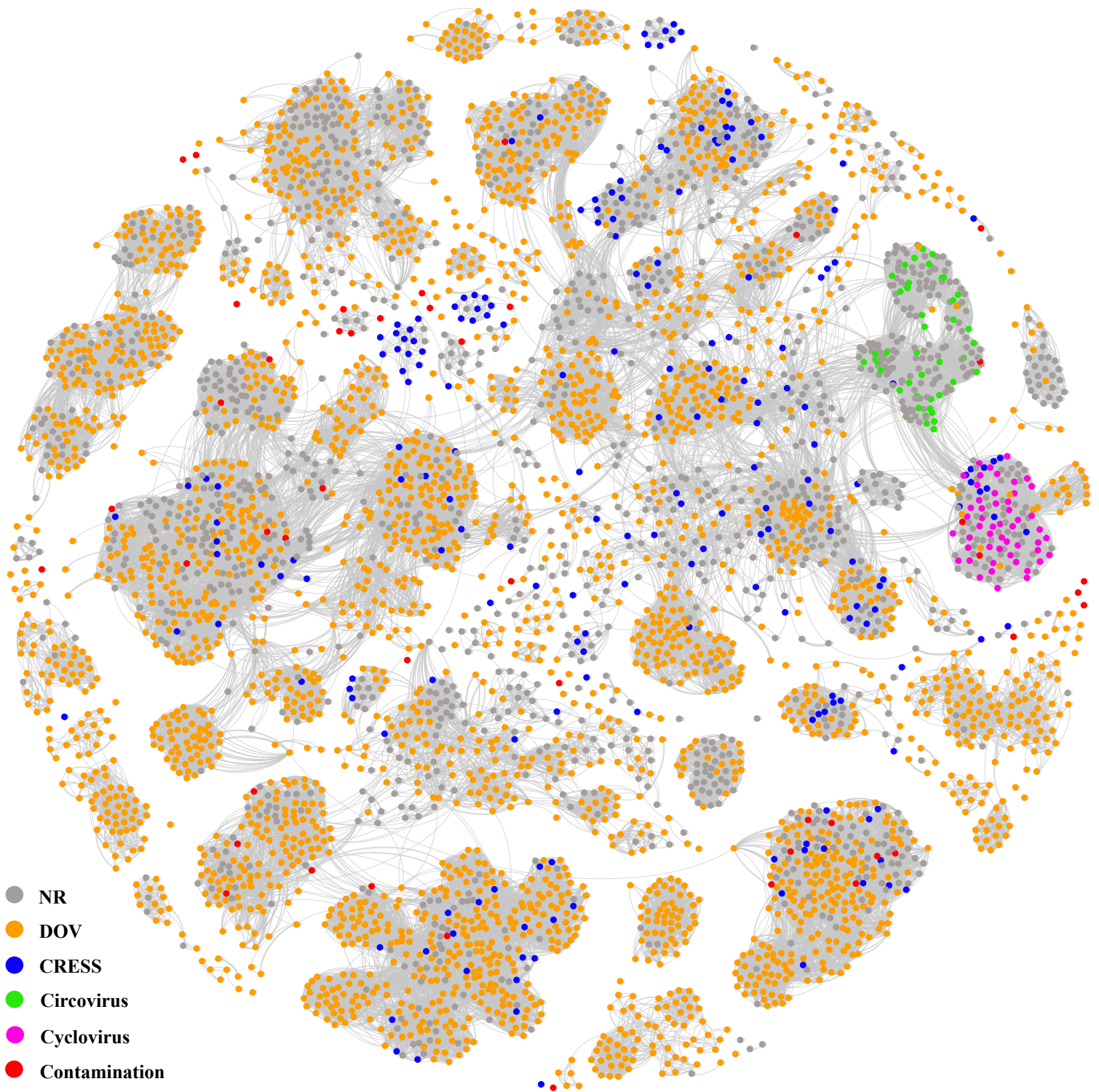

Supplement: Supplementary file 6 — Additional file 5: Figure S3. Similarity clustering of circovirus-related replicase proteins in Dataset of Oyster Virome (DOV) and NCBI nr. Dots represent different replicase sequences (n=4,716). Edges represent the score value of the Diamond BlastP results; only scores higher than 185.0 are shown. Network clustering was performed using Gephi v0.9.2 under the Fruchterman-Reingold model. Colors of dots indicate different data origins: orange, Dataset of Oyster Virome (DOV); blue, CRESS from Ashleigh et al. (2021); green, circoviruses from the International Committee on Taxonomy of Viruses (ICTV); violet, cycloviruses from the ICTV; red, contaminant sequences from Asplund et al. (2019) and Porter et al. (2021); grey, other NCBI nr sequences. [file 40168_2022_1431_MOESM5_ESM.pdf]

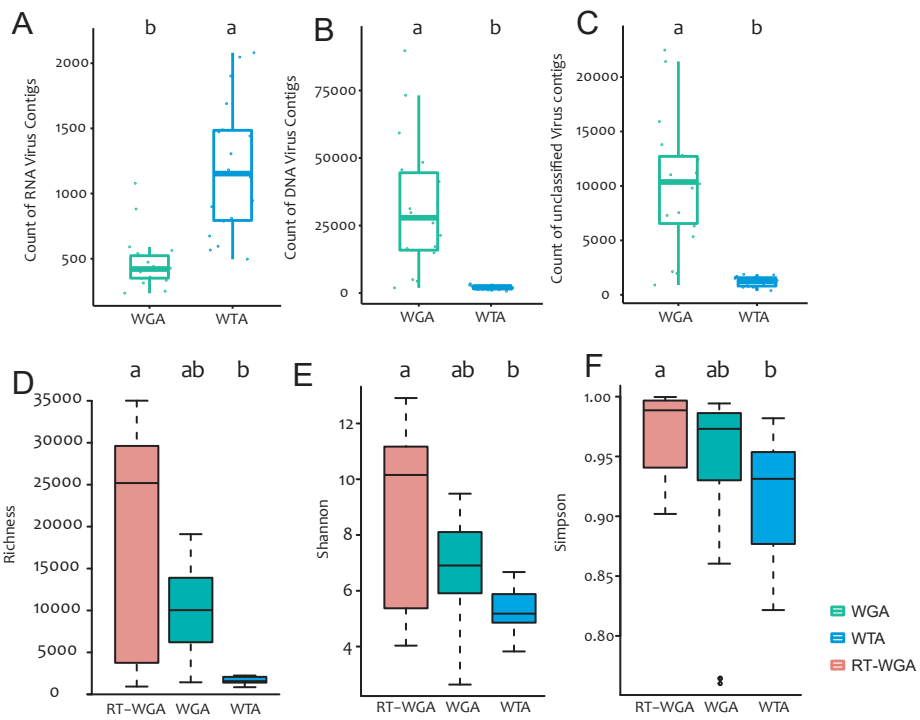

Supplement: Supplementary file 7 — Additional file 6: Figure S4. Preference of amplification strategies for the viral community and genome types. (A) Counts of RNA, (B) DNA, and (C) unclassified viral contigs (vOTU) using the WGA and WTA strategies. (D) Richness, (E) Shannon, and (F) Simpson indexes of the three amplification strategies: RT-WGA, reverse transcription and whole genome amplification; WGA, whole genome amplification; WTA whole transcriptome amplification. Different lowercase letters indicate significant differences (P <0.05; one-way ANOVAs and Tukey-Kramer post hoc comparisons). [file 40168_2022_1431_MOESM6_ESM.pdf]

A

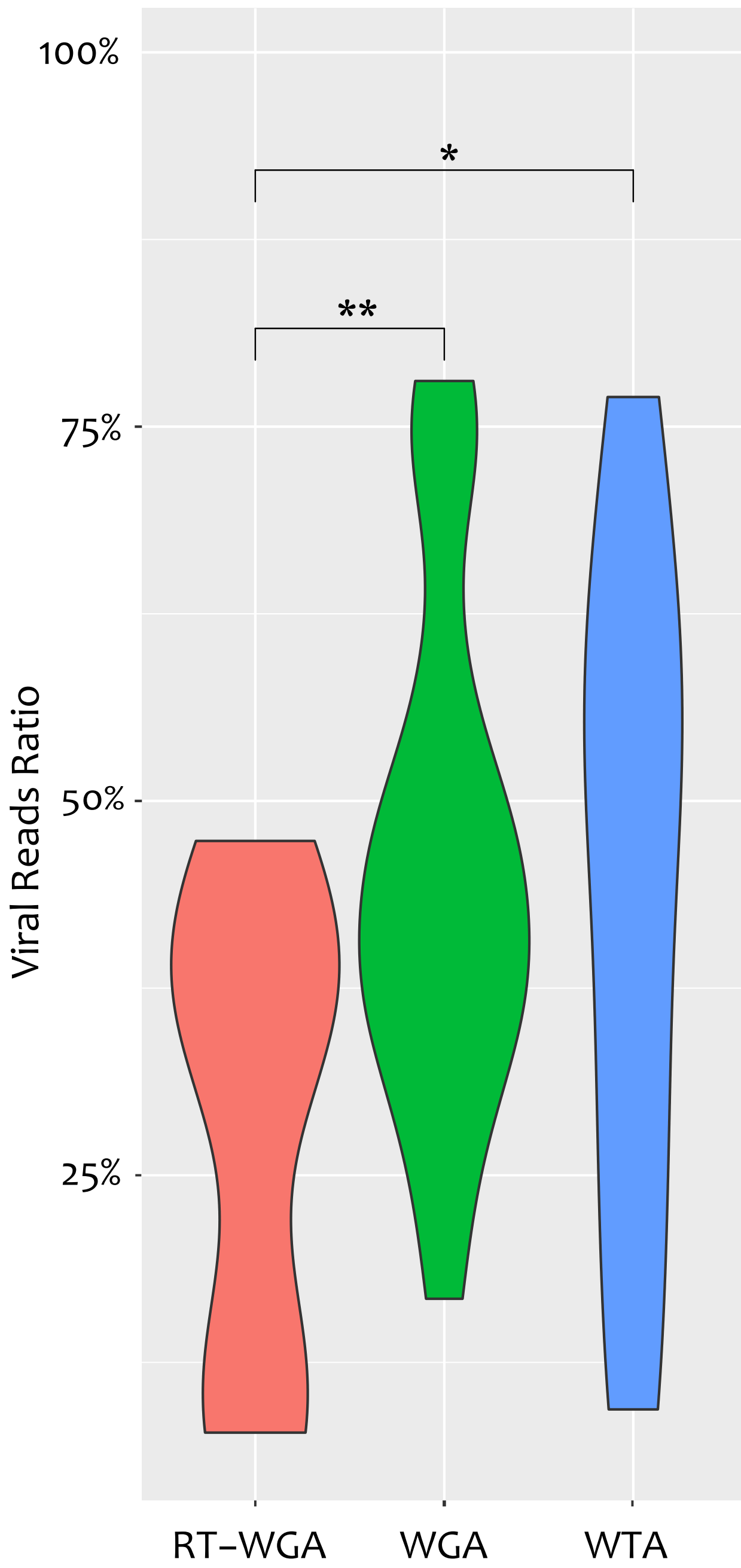

B

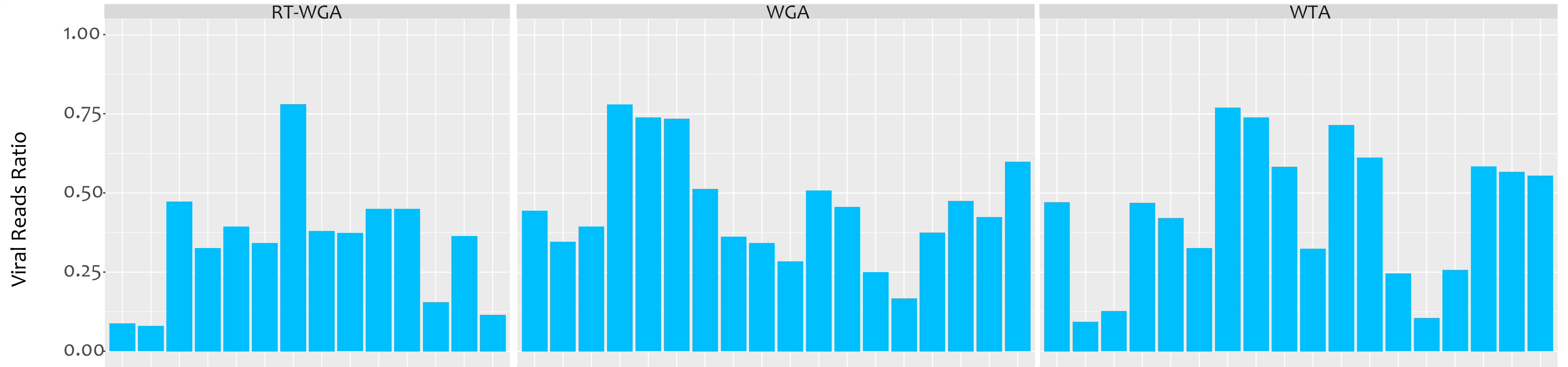

C

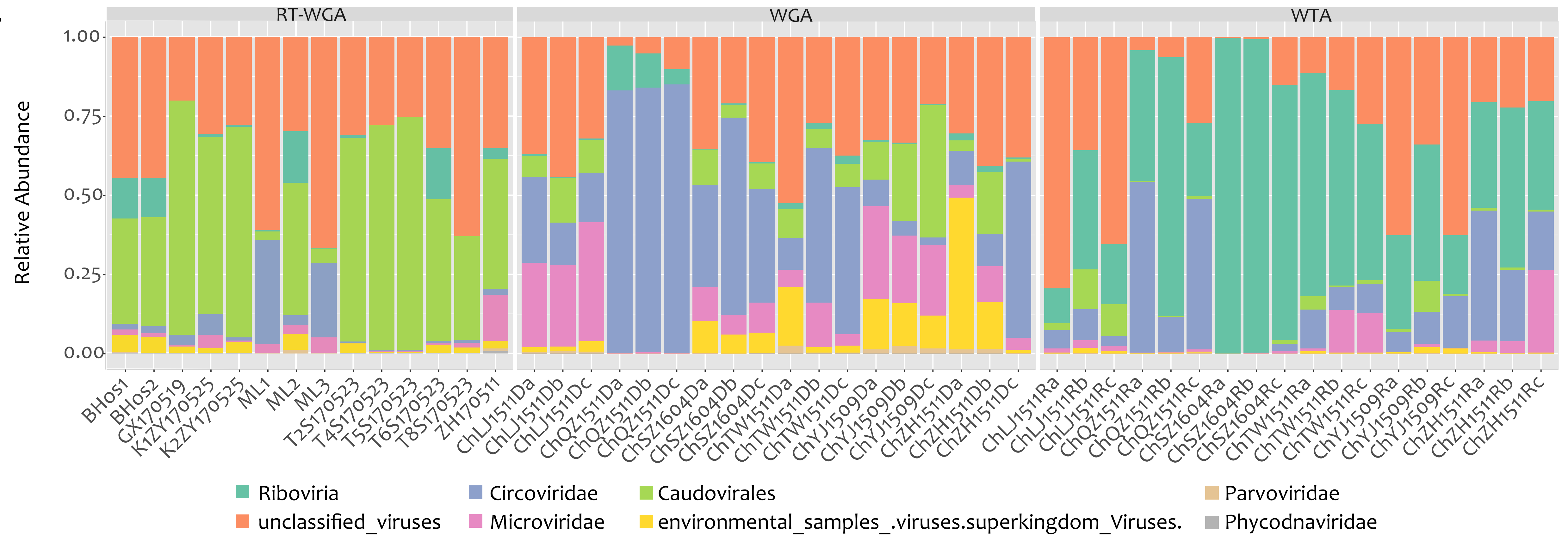

Supplement: Supplementary file 8 — Additional file 7: Figure S5. Actual and relative abundances of virus taxons in the Dataset of Oyster Virome (DOV) libraries. (A) Comparison of the viral reads ratio among three amplification groups; (B) viral reads ratio and (C) relative abundance of the taxons in the 54 DOV libraries (X-axis). Annotations are based on BLAST searches of the results of Diamond v0.9.24.125 against the NCBI nonredundant protein sequence (nr) database (release March 2021). To facilitate the display, the classifications were not unified at the same taxonomic levels. [file 40168_2022_1431_MOESM7_ESM.pdf]

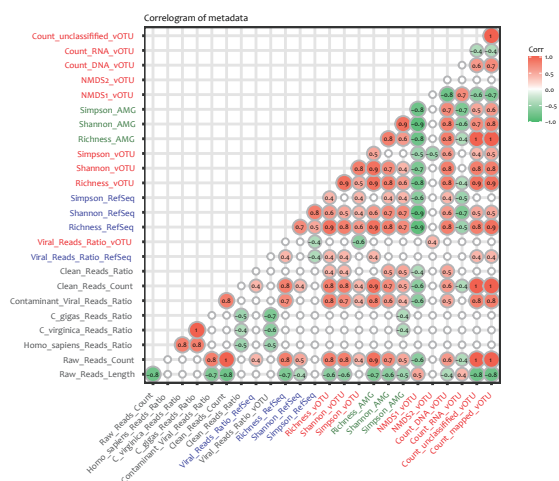

Supplement: Supplementary file 9 — Additional file 8: Figure S6. Correlation matrix of oyster viral communities. Red labels (n=10), diversity indexes, viral reads ratio, and vOTU counts based on vOTUs mapping results; black labels (n=7), quality related parameters of library construction and sequencing; blue labels (n=4), diversity indexes and viral ratio based on the reference genomes (RefSeq, GOV, and IMG/VR) mapping results; green labels (n=3): diversity indexes based on the auxiliary metabolic genes (AMGs) mapping results. [file 40168_2022_1431_MOESM8_ESM.pdf]

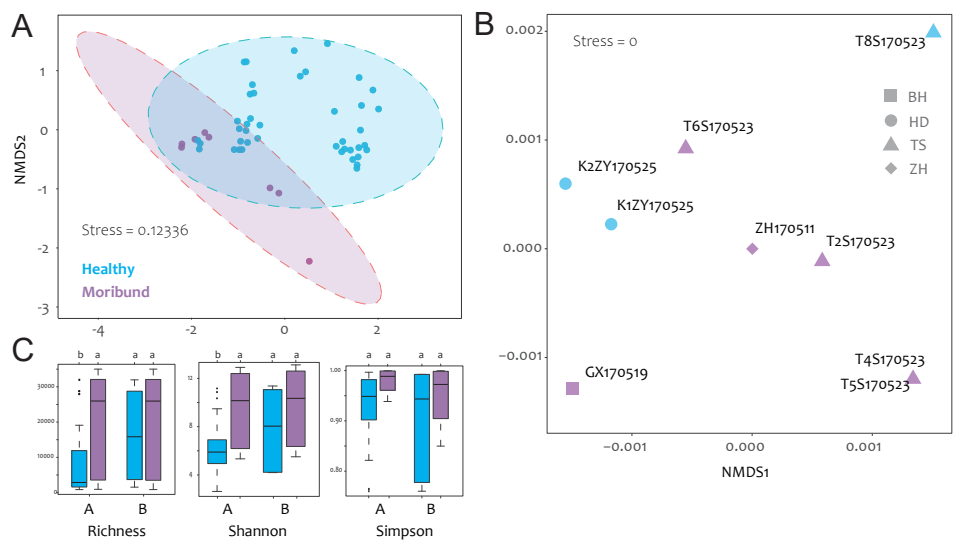

Supplement: Supplementary file 10 — Additional file 9: Figure S7. Influences of health status on the viral community in the Dataset of Oyster Virome (DOV). (A) Nonmetric multidimensional scaling (NMDS) plots of all the libraries (n=54) and (B) the seventh batch (May 2017) (n=9). (C) Comparison α-diversities (Richness, Shannon and Simpson indexes) between healthy and moribund samples corresponding to the NMDS plots in (A) and (B). Blue bar, healthy group; purple bar, moribund group. Different lowercase letters indicate significant differences (P <0.05; one-way ANOVAs and Tukey-Kramer post hoc comparisons). [file 40168_2022_1431_MOESM9_ESM.pdf]

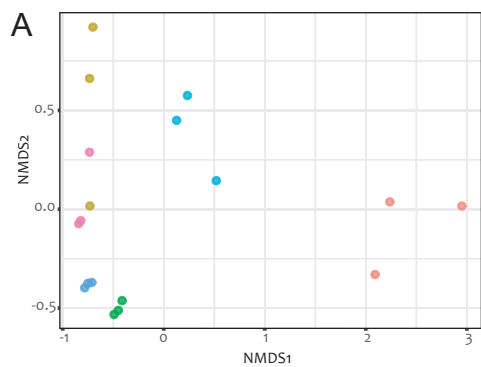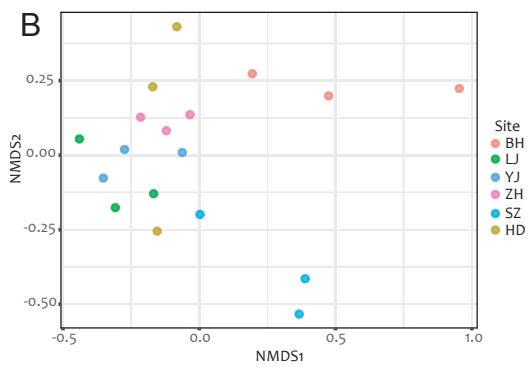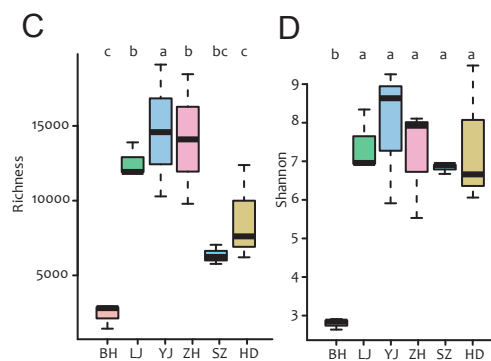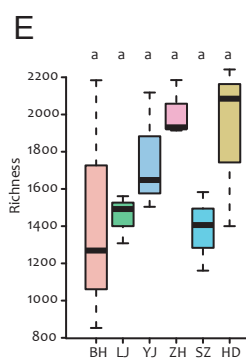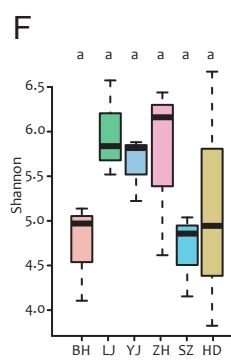

Supplement: Supplementary file 11 — Additional file 10: Figure S8. Influences of sampling sites on the viral community in the Dataset of Oyster Virome (DOV). (A, B) Nonmetric multidimensional scaling plots of different sampling sites of the WTA (A) and WGA (B) groups. (C–F) Comparisons of alpha diversity indexes among sampling sites of the WTA (C. D) and WGA (E, F) groups. The colors are used consistently in the figure. Different lowercase letters indicate significant differences (P <0.05; one-way ANOVAs and Tukey-Kramer post hoc comparisons). WGA, whole genome amplification; WTA whole transcriptome amplification. [file 40168_2022_1431_MOESM10_ESM.pdf]

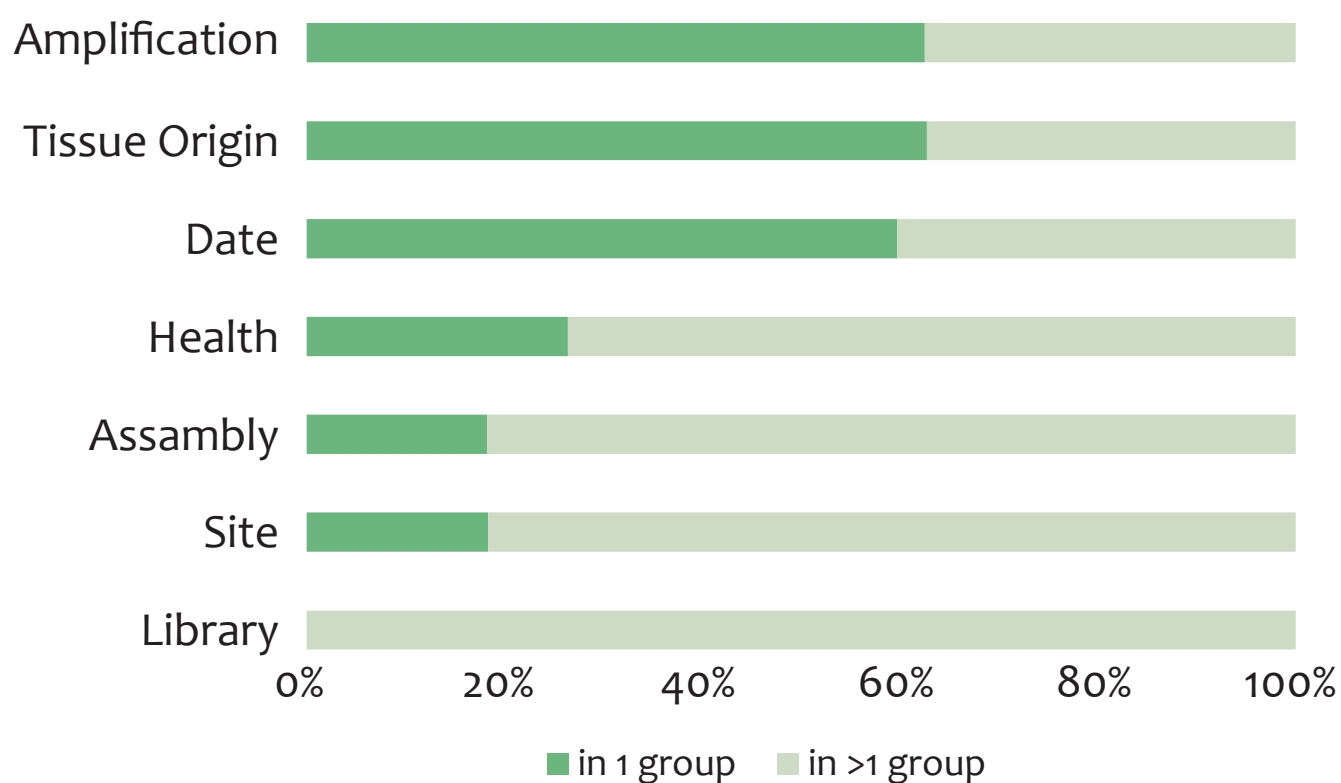

Supplement: Supplementary file 12 — Additional file 11: Figure S9. Percentage of unique viral contigs (vOTUs) (detected in only one group) under different grouping methods. [file 40168_2022_1431_MOESM11_ESM.pdf]

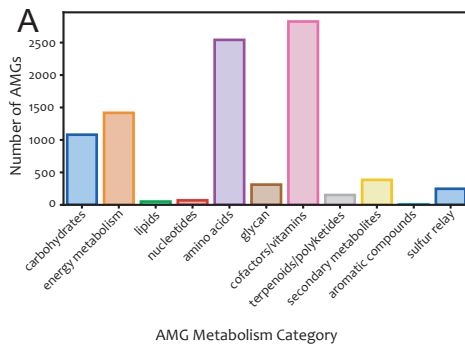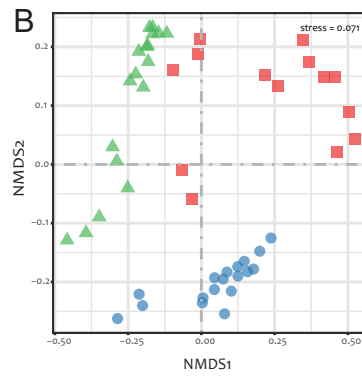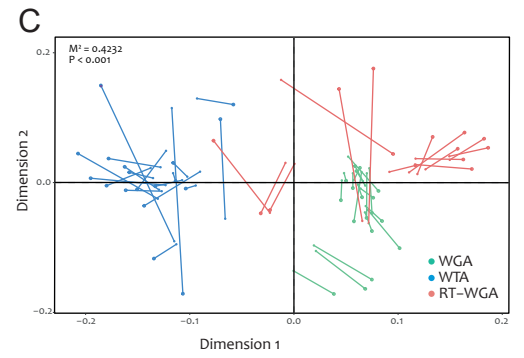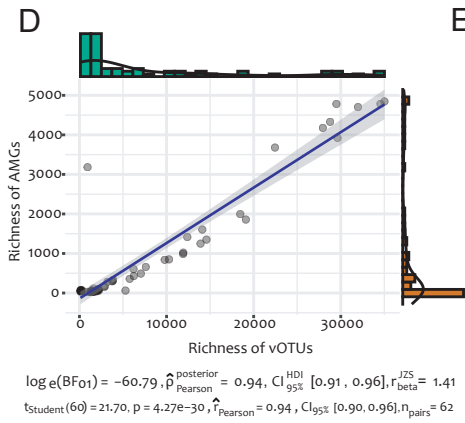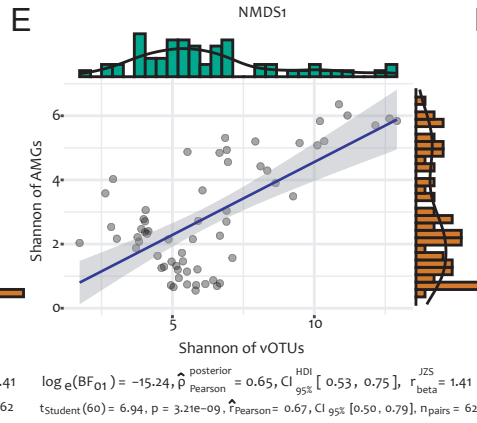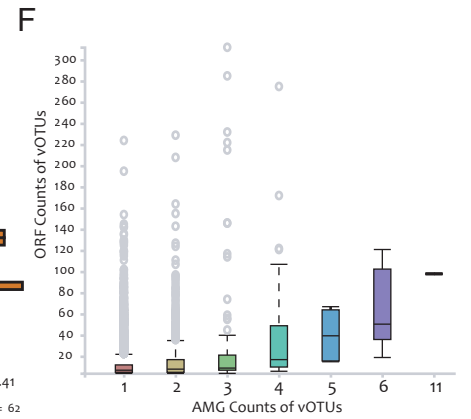

Supplement: Supplementary file 13 — Additional file 12: Figure S10. Auxiliary metabolic gene (AMG) diversity in the Dataset of Oyster Virome (DOV). (A) Number of detected AMGs assigned to different KEGG metabolic pathways. (B) Nonmetric multidimensional scaling (NMDS) plot of AMG diversity in the DOV libraries (n = 54). (C) Procrustes analysis of NMDS coordinates between the viral contig (vOTU) and AMG communities. The colors are used consistently in (B) and (C): green, WGA; blue. WTA, red. RT-WGA libraries. RT-WGA, reverse transcription and whole genome amplification; WGA, whole genome amplification; WTA whole transcriptome amplification. (D, E) Correlations and linear correlation curves of the Richness (D) and Shannon (E) indexes between vOTUs and AMGs. (F) Correlation between AMG and open reading frame (ORF) counts on the same vOTU. [file 40168_2022_1431_MOESM12_ESM.pdf]
